# Serum IgM against SARS-CoV-2 correlates with in-hospital mortality in severe/critical patients with COVID-19 in Wuhan, China

**DOI:** 10.18632/aging.103417

**Published:** 2020-07-06

**Authors:** Xintian Liu, Xuan Zheng, Bo Liu, Mingxiang Wu, Zhenlu Zhang, Gangcheng Zhang, Xi Su

**Affiliations:** 1 Intensive Care Unit, Wuhan Asia General Hospital, Wuhan 430050, China; 2 Cardiac Center, Wuhan Asia Heart Hospital, Wuhan 430022, China; 3 Department of Cardiology, Wuhan Asia Heart Hospital, Wuhan 430022, China; 4 Department of Clinical Laboratory, Wuhan Asia General Hospital, Wuhan 430050, China

**Keywords:** severe acute respiratory syndrome coronavirus 2, COVID-19, intensive care, antibody, in-hospital mortality

## Abstract

Severe/critical patients with coronavirus disease 2019 (COVID-19) have become the central issue in the current global pandemic due to their high mortality rate. However, the relationship between antibody response and clinical outcomes has not been well described in this group. We conducted a single-center, retrospective, cohort study to investigate the relationship between serum immunoglobulin G (IgG) and IgM and clinical outcomes in severe/critical patients with COVID-19. Seventy-nine severe/critical patients with COVID-19 admitted in Wuhan Asia General Hospital in Wuhan, China during January 22, 2020 to March 6, 2020 were included. Serum antibodies were measured at day 25 (SD, 7) post illness onset. The median IgG titer was 113 (IQR 81-167) AU/ml, and IgM titer was 50 (IQR, 23-105) AU/ml. Patients whose IgM titer ≥ 50 AU/ml had higher in-hospital mortality (p=0.026). IgM titer ≥ 50 AU/ml was also correlated with higher incidences of Acute Respiratory Distress Syndrome (ARDS) and sepsis shock. Antibody remeasurements were performed in 42 patients, where IgM titer declined significantly in survivors (p*=*0.031). Serum IgM titer changes according to the COVID-19 progression. The severe/critical patients with COVID-19 have a higher risk of clinical adverse events when IgM titer ≥ 50 AU/ml. Further decreasing of IgM could imply a better outcome in severe/critical cases.

## INTRODUCTION

Coronavirus disease 2019 (COVID-19) was first reported in Wuhan, China in December 2019. The highly contagious pneumonia caused by severe acute respiratory syndrome coronavirus 2 (SARS-CoV-2) soon spread all over the country, and has become a global pandemic [1–4]. Patients infected by SARS-CoV-2 might present from asymptomatic to critical illness with respiratory failure and multi-organ dysfunction, therefore, the disease was categorized into 4 types based on the disease state: mild, moderate, severe, and critical [5, 6]. Severe/critical patients with COVID-19 contributed only 4~15% to overall infected population in different countries [7, 8], however, attentions have been paid to them not only because of their rapid progression in disease, but also due to the greater difficulties in treatment and higher mortality rate [7, 9, 10].

Antibody response in human might be activated at early stage of infectious disease, then be kept stable for a long time. Specific serum immunoglobulin G (IgG) and IgM against SARS-CoV or Middle East Respiratory Syndrome-coronavirus (MERS-CoV) became detectable in patients as early as 11-15 days post illness onset [11, 12]. Similar changes were observed in patients with COVID-19 as IgM and IgG could be detected on 5-14 days after symptom onset [13]. Additionally, the titers of IgM and IgG were significantly correlated with viral load in patients infected by SARS-CoV-2 in a recent finding [14], which promoted the hypothesis that specific antibody against virus might be associated with disease progression in COVID-19. However, reports on clinical profiles of antibody response in severe/critical patients with COVID-19 are scarce.

Hereby, we investigated the serum titers of specific antibodies, IgG and IgM, in severe/critical patients with COVID-19 to explore the association between serum antibody titers and the clinical adverse events in those patients.

## RESULTS

### Characteristics of the patients

A total of 105 severe/critical patients with COVID-19 admitted to Wuhan Asia General Hospital from 2020.01.22 to 2020.03.06 were enrolled, Of which, 23 were excluded due to the incomplete data, 3 due to negative in antibody measurements. Therefore, 79 patients were reviewed in final analysis, whose mean age was 63 (SD 13) years. Seven (9%) patients were smokers, and comorbidities included 5 (6%) chronic obstructive pulmonary disease (COPD), 31 (39%) hypertension, 13 (16%) diabetes, 6 (8%) coronary artery disease (CAD), and 2 (3%) chronic kidney disease (CKD). The most common symptoms were fever in 64 (81%) patients, cough in 57 (72%), dyspnea in 49 (62%), and fatigue in 44 (56%). The average time from illness onset to admission was 12 days (SD, 6). All patients had significantly change on lung computerized tomography (CT).

### Antibody response and in-hospital mortality

**Figure 1:**
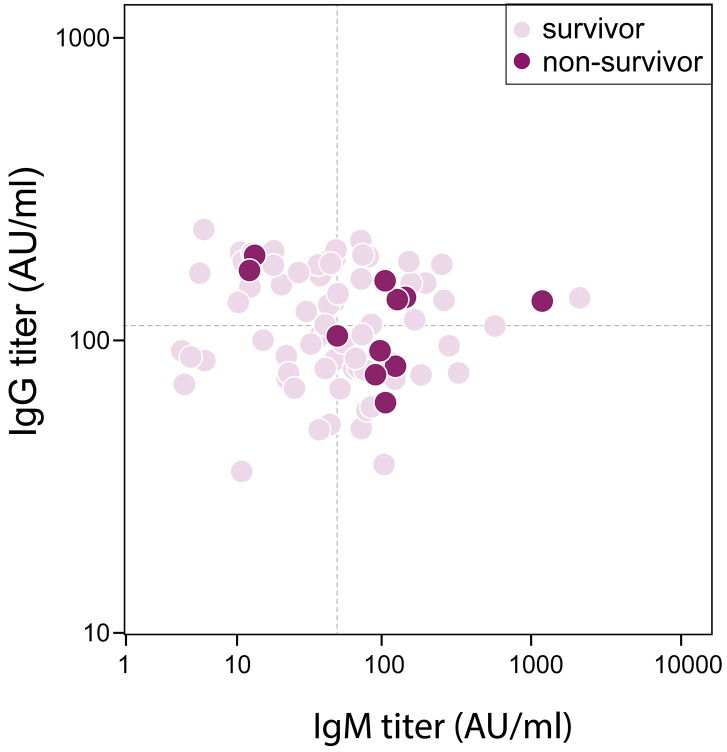
**Correlation between Antibody titer and in-hospital mortality in severe/critical patients with COVID-19.** Dash lines represent median value as cutoff in IgG (113 AU/ml) and IgM (50 AU/ml) respectively.

**Figure 2:**
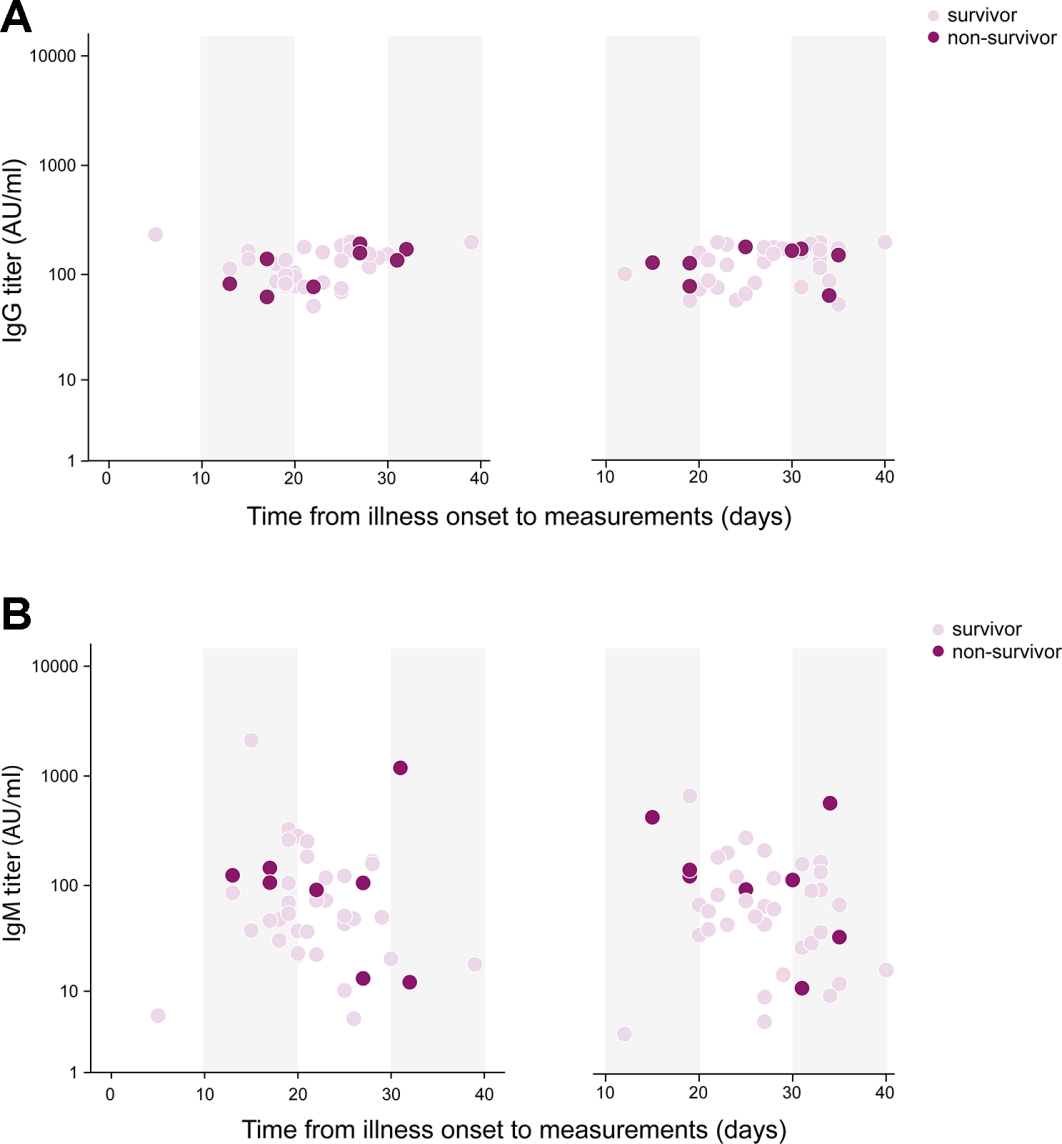
**Temporal profile of serum antibodies in severe/critical patients with COVID-19.** 42 patients had two antibody measurements on day 25 (SD, 7) and on day 27 (SD, 6) post illness onset respectively. (**A**) IgG titer remained stable during two measurements in both survivors and non-survivors. (**B**) Change of IgM titer in survivors showed a significantly decreasing (-4 [IQR -14-0], P=0.031), but that in non-survivors didn’t show statistical difference (3 [IQR -19-29], P=0.779).

Eleven (14%) patients died during hospitalization, who were older than survivors (73 [SD 9] vs 61 [SD 2], *P*=0.002). There were 16 (20%) Acute Respiratory Disease Syndrome (ARDS) and 11 (14%) septic shock happening during hospitalization. Patients had their measurements of serum antibody against SRAS-CoV-2 on day 13 (SD, 7) post admission when tests were available, which was 25 (SD, 7) days after illness onset. The median IgG titer was 113 (IQR, 81-167) AU/ml, and that of IgM was 50 (IQR, 23-105) AU/ml. The difference of IgG titer between survivors and non-survivors was trivial (113 [IQR, 81-167] vs 135 [IQR, 82-158] AU/ml, *P=*0.887), however, IgM titer was significantly increased in non-survivor when comparing with survivors (106 [IQR, 50-128] vs 48 [IQR, 22-84] AU/ml, *P=*0.049) (Figure 1). Forty-two patients had antibody remeasurements 5 (SD, 3) days later. The median IgG titer was 150 (IQR 88-179) AU/ml at 2^nd^ time, and that of IgM was 66 (IQR 32-133) AU/ml. IgG titer remained stable during two measurements in both survivors and non-survivors. Change of IgM titer in survivors showed a significantly decreasing (-4 [IQR -14-0], *P=*0.031), but that in non-survivors didn’t show statistical difference (3 [IQR -19-29], *P*= 0.779) (Figure 2).

### Serum IgM and clinical outcomes

We further divided patients into two groups using median serum IgM titer as cutoff. Clinical characteristics, such as age, gender, comorbidity, symptoms, time intervals, and vital signs at admission, were similar between the two groups (Table 1). Disease severity was quite different, as a higher incidence of critical cases was seen in the high IgM group (p*=*0.006) (Table 1). Laboratory measurements presented differently between groups (Table 2). All patients received basic therapy as well as specific treatment based on their disease progression in hospital. More Intensive medical supports were applied in patients whose IgM titer ≥ 50 AU/ml (Table 3).

**Table 1:** Clinical characteristics of patients with different IgM titers.

	**IgM < 50 AU/ml (n=39)**	**IgM ≥ 50 AU/ml (n=40)**	***P***
Age, years	64±11	61±14	0.315
Men	25(64)	25(63)	0.883
Current smoker	5(13)	2(5)	0.221
Comorbidity			
Chronic obstructive lung disease	3(8)	2(5)	0.623
Hypertension	17(44)	14(35)	0.434
Diabetes	7(18)	6(15)	0.724
Coronary heart disease	4(10)	2(5)	0.378
Chronic kidney disease	0(0)	2(5)	0.494
Symptoms			
Fever	30(77)	34(85)	0.360
Cough	28(72)	29(73)	0.944
Sputum	15(38)	11(28)	0.300
Myalgia	1(3)	5(13)	0.201
Fatigue	22(56)	22(55)	0.900
Diarrhoea	6(15)	6(15)	0.962
Dyspnea	25(64)	24(60)	0.707
Time from illness onset to hospital admission, days	10(7-14)	12(10-14)	0.172
Time from illness onset to first antibody detection, days	26(21-31)	23(19-29)	0.183
Time from hospital admission to first antibody detection, days	13(9-21)	11(7-15)	0.153
Vital signs on admission			
Temperature, °C	36.9±0.6	36.9±0.9	0.774
Systolic pressure, mmHg	129±18	128±18	0.857
Diastolic pressure, mmHg	78±12	76±9	0.461
Heart rate, beats/min	91±18	87±14	0.275
Disease severity state			0.003
Severe	36(92)	26(65)	
Critical	3(8)	14(35)	

Data are mean ± SD, median (IQR) or n (%). IgM = Immunoglobulin M.

**Table 2:** Laboratory measurements of patients with different IgM titers.

	**IgM < 50 AU/ml (n=39)**	**IgM ≥ 50 AU/ml (n=40)**	***P***
Arterial blood gas analysis			
PH	7.38±0.06	7.40±0.05	0.136
PaCO2, mmHg	44±7	42±8	0.277
PaO2, mmHg	59±6	56±7	0.044
SaO2, %	91±4	89±4	0.039
White blood cell count, ×10^9^/L	6.9±3.0	7.1±2.8	0.777
Neutrophil count, ×10^9^/L	5.5±2.9	5.8±2.9	0.608
Lymphocyte count, ×10^9^/L	0.9±0.4	0.9±1.0	0.800
Haemoglobin, g/L	126±15	126±19	0.812
Platelet count, ×10^9^/L	249±118	228±87	0.369
ALT, U/L	24(18-44)	39(16-63)	0.161
Albumin, g/L	34±4	32±5	0.010
Creatinine, μmol/L	86±26	83±38	0.730
Prothrombin time, s	12.0±0.8	12.4±1.2	0.085
Fibrinogen, g/L	5.0±1.9	5.2±1.7	0.623
D-dimer, mg/L	0.95(0.44-2.59)	1.81(0.77-9.06)	0.020
Cardiac troponin T, pg/ml	10(6-18)	12(8-20)	0.666
NT-proBNP, pg/ml	80(59-252)	264(73-590)	0.031
C-reactive protein, mg/L	40(12-107)	69(27-126)	0.119
IL-6, pg/mL	17(6-70)	42(12-119)	0.141
TNF-α, pg/mL	11(8-17)	9(5-12)	0.111

Data are mean ± SD or median (IQR). IgM = Immunoglobulin M. PH = Pondus Hydrogenii. PaCO2 = partial pressure of carbon dioxide. PaO2 = partial pressure of oxygen. SaO2 = arterial oxygen saturation. ALT = alanine aminotransferase. NT-proBNP = N-terminal pro-brain natriuretic peptide. IL-6=interleukin-6. TNF-α = tumor necrosis factor-α.

**Table 3:** Treatments and outcomes of patients with different IgM titers

	**IgM < 50 AU/ml(n=39)**	**IgM ≥ 50 AU/ml(n=40)**	***P***
Drugs			
Antiviral treatment	36(92)	38(95)	0.675
Antibiotics	36(92)	39(98)	0.359
Corticosteroids	16(41)	32(80)	<0.001
Chinese traditional medicine	39(100)	39(98)	1.000
Oxygen inhalation	38(97)	38(95)	0.571
Mechanical ventilation	3(8)	14(35)	0.003
Non-invasive	3(8)	13(33)	0.006
Invasive	0(0)	9(23)	0.002
Other advanced supportive therapy	1(3)	4(10)	0.175
IABP	0(0)	1(3)	0.320
CRRT	1(3)	4(10)	0.175
ECMO	0(0)	2(5)	0.157
Outcomes			
ARDS	2(5)	14(35)	0.001
Septic shock	2(5)	9(23)	0.026
In-hospital mortality	2(5)	9(23)	0.026
Hospital length of stay, days	29(21-30)	29(19-31)	0.941

Data are median (IQR) or n (%). IgM = Immunoglobulin M. IABP = intra-aortic balloon pump. CRRT = continuous renal replacement therapy. ECMO = extracorporeal membrane oxygenation. ARDS = Acute Respiratory Distress Syndrome.

## DISCUSSION

In this retrospective cohort study, IgG and IgM against SARS-CoV-2 in severe/critical patients with COVID-19 were profiled, and relationship between antibody titers and outcomes was also assessed. Specifically, compared with survivors, IgM titer increased in non-survivors while IgG remained unchanged when measurements were performed on 25 (SD, 7) days after illness onset. IgM further decreased in survivors when taking remeasurement 5 (SD, 3) days later. Accompanied by significantly changes in laboratory measurements, more critical cases were seen in patients with IgM titer ≥ 50 AU/ml. Higher frequencies of applying corticosteroids and mechanical ventilation were also observed in patients with IgM titer ≥ 50 AU/ml.

Pneumonia caused by SARS-CoV-2, which was later known as COVID-19, occurred in Wuhan, China in December 2019 [1, 15]. The estimated reproductive number rose from 2.2 to 3.28 [14], and overall mortality rate was around 2-4% [16–18], which might be still increasing as more than one million patients have been confirmed infection, and new deaths are reported globally. Nearly 80% of patients with COVID-19 might present only mild or moderate symptoms, such as fever, and cough [8, 19], however, more than 50% death could be seen in severe/critical cases [7, 20]. Similar to previous studies, non-survivors in our study were older than survivors. There were no differences in comorbidities between survivors and non-survivors in our study, probably due to the variation in the spectrum of underlying diseases. In-hospital mortality (14%) in our study was lower than that in other reports, nonetheless, at least 5 folds higher mortality in severe/critical patients, again, strengthened that great efforts should be paid on this group.

Serum IgM is the first protein producing in human in response to the exposure to an antigen, such as bacterial, virus, and others. IgM titer could increase in hours to respond antigen attack followed by degradation in weeks. Being a secondly important antibody, IgG would be activated in a moderate but long-lasting way. It might slowly rise in weeks after recognizing antigen, and reach a plateau for years. Guo et al. examined 208 samples from confirmed and suspected patients with COVID-19. Specific antibodies could be positive as early as day 1 after illness onset. For most patients, IgM appeared at day 5 and became stable at days 15-21 after increasing at day 8. IgG showed same change as IgM at acute phase but continued its rising until plateau at day 21 [13]. Our patients had their antibody measurement on day 25 (SD7), and repeated on day 27 (SD6). Despite of the stable levels in IgG and IgM, our measurements were performed later than other studies. We believed the results were still robust because the measurements were performed at the time when both IgG and IgM were in plateau according to previous studies [21], and the IgG and IgM titers remained high and detectable in our study. Moreover, we observed IgM might decrease on day 27 (SD 6) if patients recovered. As Mo et al mentioned in their study, IgM against SARS-CoV declined much earlier than IgG [22]. The decreasing of IgM against SARS-CoV-2 in survivors from our study might be a natural change of IgM in COVID-19. On the other hand, To et al. investigated the correlation between serum antibody response and viral load. They found IgG and IgM titers were highly correlated with viral load in patients with COVID-19, which might explain why our patients had a recover in their illness in consistent with IgM decreasing [14]. One thing might be noticed, there were 10 patients having negative molecular tests in our study, even though they presented critical illness. Similar findings were seen in the study by Zhang et al. They observed positive rate in molecular tests might be reducing as time from illness onset prolonged, while IgG and IgM titers were stable in all patients [23]. The reasons for this were discussed before: viral RNA might vary from oral swabs to anal swabs; mismatch in the detection probes; fluctuation in viral load unparalleled with illness progression [24, 25].

Efforts have been made to distinguish patients at high risk of mortality. Studies proposed age, comorbidities, CT imaging, and other parameters, which showed differences in survivors and non-survivors [26, 27], to predict risks in patients with COVID-19. Nonetheless, we didn’t find many differences between survivors and non-survivors in our study. Severe and critical illness in our patients might eliminate the influence by other factors. On the contrary, our study supported the clinical application of serum IgM in severe/critical patients with COVID-19 for risk stratification. Significantly higher mortality rate was seen in patients when their serum IgM was higher than 50 AU/ml. Additionally, serial changes in IgM titer also helped to follow the disease progression in patients with poor prognosis.

Our study showed that advanced supportive treatment together with combination therapy were more applied in patients with high mortality. The high levels of IgM in our patients might indicate a disease worsening despite of the treatment. Treatment strategy was proposed based on the disease stage, however, no evidence had been shown to be most specific to COVID-19 [28]. Patients might show different response to corticosteroids [29, 30]. Although patients admitted into ICU required more medical treatments, the effect of advanced support seemed to be controversial in critical patients [31, 32]. The ideally strategy to treat viral pneumonia has always been remove the virus as soon as possible. The antivirus effect by Remdesivir in patient and cells might bring hope in further treatment [33, 34].

There were some limitations in our studies. Firstly, there were only 79 patients included in our study. The small size of study population might bring bias to data distribution. Further study should involve more patients to investigate the clinical profile of antibody response. Secondly, our antibody measurements started on 25 days post illness onset. The late measurements missed early change of antibody in patients. New studies might consider a broader interval to cover more changes. Thirdly, we focused on in-hospital mortality for severe/critical patients. However, there were reports that patients might have disease progression after discharge [24]. We might follow-up our patients for a longer time to see the relationship between antibody titer and their prognosis.

## CONCLUSIONS

Our study demonstrated the dynamic change of antibody titer in consistent with disease progression. A higher risk of in-hospital mortality was seen in severe/critical patients of COVID-19 when their IgM titer ≥ 50 AU/ml. Further decreasing of IgM could imply a better prognosis in severe/critical patients. Serial measurements of serum antibody provide comprehensive evaluation to the process of COVID-19.

## MATERIALS AND METHODS

### Study design and patients

A retrospective cohort study was conducted in Wuhan Asia General Hospital, Wuhan, China to investigate the clinical profile of serum antibodies against SARS-CoV-2 in severe/critical patients with COVID-19. The study protocol was reviewed and approved by the Ethics Committee of Wuhan Asia General hospital with a waiver of informed consent (WAGHMEC-KY-2020007). Personal information of patients was re-identified before analysis.

A total of 105 severe/critical patients with COVID-19 admitted in Wuhan Asia General hospital between 2020.01.22 and 2020.03.06 were reviewed. COVID-19 was diagnosed according to the Chinese management guideline for COVID-19 (version 7.0) [6]. New laboratory criteria of COVID-19-specific IgM and IgG positive, and 4 folds increasing of COVID-19-specific IgG titer in recovery period were added in guideline 7.0 [6]. Severe patients with COVID-19 met any of the followings: (1) Shortness of breath, respiratory rate ≥ 30 times per minute; (2) Oxygen saturation ≤ 93% at rest; (3) Alveolar oxygen partial pressure/fraction of inspiration O2 (PaO2/FiO2) ≤ 300 mmHg (1mmHg=0.133kPa). Critical patients had any of the conditions: (1) Respiratory failure requiring mechanical ventilation; (2) Shock; (3) Patients combined with other organ failure needed intensive care unit (ICU) monitoring and treatment [6]. Fever was defined as axillary temperature greater than 37·3°C.

### Data collection

Clinical data including age, gender, vital signs, comorbidity were collected from medical records at admission. Laboratory biomarkers such as IgG titer, IgM titer, blood gas analysis, white blood cell count (WBC), alanine aminotransferase (ALT), D-dimer, and N-terminal prohormone of brain natriuretic peptide (NT-proBNP) were also collected. Specifically, serum IgG and IgM that against SARS-CoV-2 nucleocapsid protein and envelop protein were measured by chemiluminescence immunoassay (CLIA) in automatic system when it was available on February 18, 2020. Antibody titer > 10 AU/ml was taken as positive. All blood tests were analyzed in fresh blood and determined by standard quantitative assay techniques in our Department of Clinical Laboratory according to the manufacturer’s protocol.

### Outcomes

The primary outcome was in-hospital mortality. The secondary outcomes included ARDS related to SARS-CoV-2 infection and sepsis shock secondary to COVID-19. ARDS was diagnosed according to the Berlin Definition [35]. Sepsis shock was defined according to the 2016 Third International Consensus Definition [36].

### Statistical analysis

Data are shown as number for categorical data, and mean ± standard deviation or median with interquartile range (IQR) as appropriate for continuous data. Data were compared with student t test or Wilcoxon signed-rank test for continuous variables depending on the normality of their distributions and with the χ2 test for categorical variables. Comparison between the first and second antibody titer is performed by paired samples Wilcoxon test. A two-side *P* < 0.05 was considered as statistic significant. All statistical analyses were performed with SPSS 23.0 (IBM Corp, Armonk, NY).
